# Form-Stable Phase Change Materials Based on Eutectic Mixture of Tetradecanol and Fatty Acids for Building Energy Storage: Preparation and Performance Analysis

**DOI:** 10.3390/ma6104758

**Published:** 2013-10-22

**Authors:** Jingyu Huang, Shilei Lu, Xiangfei Kong, Shangbao Liu, Yiran li

**Affiliations:** School of Environment Science and Technology, Tianjin University, Tianjin 300072, China; E-Mails: hjy0104@126.com (J.H.); nesta7603@126.com (X.K.); happy2012liu@163.com (S.L.); lyr@tju.edu.cn (Y.L.)

**Keywords:** form-stable PCM, tetradecanol, eutectic mixtures, HDPE, EVA, building energy storage

## Abstract

This paper is focused on preparation and performance analysis of a series of form-stable phase change materials (FSPCMs), based on eutectic mixtures as phase change materials (PCMs) for thermal energy storage and high-density polyethylene (HDPE)-ethylene-vinyl acetate (EVA) polymer as supporting materials. The PCMs were eutectic mixtures of tetradecanol (TD)–capric acid (CA), TD–lauric acid (LA), and TD–myristic acid (MA), which were rarely explored before. Thermal properties of eutectic mixtures and FSPCMs were measured by differential scanning calorimeter (DSC). The onset melting/solidification temperatures of form-stable PCMs were 19.13 °C/13.32 °C (FS TD–CA PCM), 24.53 °C/24.92 °C (FS TD–LA PCM), and 33.15 °C/30.72 °C (FS TD–MA PCM), respectively, and latent heats were almost greater than 90 J/g. The surface morphologies and chemical stability of form-stable PCM were surveyed by scanning electron microscopy (SEM) and Fourier-transform infrared (FT-IR) spectroscopy, respectively. The thermal cycling test revealed that the thermal reliability of these three form-stable PCMs was good. Thermal storage/release experiment indicated melting/solidification time was shortened by introducing 10 wt % aluminum powder (AP). It is concluded that these FSPCMs can act as potential building thermal storage materials in terms of their satisfactory thermal properties.

## 1. Introduction

With the rapid development of construction industry, more and more lightweight building materials are applied in high-rise buildings. Because of the low thermal capacity of lightweight materials, indoor temperature tends to fluctuate under climate change. Phase change materials (PCMs) with their high heat storage capacity have been considered as potential latent storage materials widely studied in building thermal storage [1]. PCMs absorb redundant heat in daytime, the stored thermal energy release into indoors at night. Through this thermal energy circulation, the indoor temperature maintains in a relative comfortable temperature range. The traditional PCMs are mainly divided into organic materials and inorganic materials. Several reviews have concluded a large category of candidate materials for latent heat storage [1,2,3,4]. Organic materials such as fatty acids and their eutectic mixtures have superior properties over inorganic materials like litter super cooling, high latent heat, less volume change, good thermal, and chemical stability after repeated cycles [5,6,7,8]. In recent years, the studies of mixtures of fatty acids and alcohols have caught more attention [9,10,11,12]. Zeng [11] studied thermal properties and thermal conductivity of a series of palmitic acid (PA) and tetradecanol (TD) mixture. The phase change temperature of PA-TD eutectic mixture was 29.00 °C, which can be viewed as a new PCM. Zuo [12] investigated the thermal properties of lauric acid (LA)/1-tetradecanol binary system by differential scanning calorimeter (DSC). The eutectic melting temperature was 24.33 °C under common building environment temperatures of 10–40 °C.

However, PCM cannot be directly applied in building envelop due to lager leakage and excitant odor of PCM. Many scholars have developed a kind of form-stable PCMs, which encapsulated PCM in polymeric network structures. Form-stable PCMs prevent the leakage of liquid PCM when it is in a state of melting [8,13,14,15,16]. Inaba prepared a form-stable PCM with mass percentage of 74 wt % paraffin/26 wt % high-density polyethylene (HDPE), and analyzed thermophysical properties, such as thermal conductivity, latent heat, specific heat, and density. Furthermore, correlation equations between mass percentage and the aforementioned were put forward [17]. Sari investigated thermal properties of form-stable paraffin/HDPE composites, which consist of two different kinds of paraffin. The maximum mass percentage of each paraffin, in two different PCM composites, was as high as 77%. In addition, with the addition of 3 wt % expanded and exfoliated graphite, the thermal conductivity of two composites increased about 14% and 24%, respectively [16]. From 2005 to 2009, Cai [13,18,19] published a series of literature concerning preparation, properties analysis of different form-stable PCMs, which consisted of paraffin as latent heat storage material. HDPE as supporting material have been studied for a long time. While HDPE is easy to aging and cracking after long-term recycling, thus, it is not suitable to be applied in buildings, which should be used as long as 30 years. In order to solve this problem, HDPE has to be modified. Ethylene-vinyl acetate (EVA) is a kind of modified polyethylene material with good flexibility, anti-aging, and resistance to environmental stress cracking. A new HDPE-EVA polymer blend including different structures of ethylene polymers could be achieved by a mechanical blending method. This polymer was homogeneous and continuous structure on macroscopic. The molecular micelles of HDPE and EVA uniformly dispersed. This polymer can possess the advantage of HDPE and EVA.

In addition, in order to overcome low thermal conductivity of organic materials as PCM, Liu [20] summarized several mainstream thermal enhancement techniques for high temperature phase change thermal storage systems. Adding high thermal conductive materials, such as metal [aluminum powder (AP), Ag] and expanded graphite, into PCM produced effective thermal storage materials [16,21,22,23,24,25]. According to thermal storage and release tests of pure hexadecane and aluminum/hexadecane composite, Darkwa concluded that thermal response rate especially heat flux rate was accelerated by introducing AP. Thermal conductivity of aluminum/hexadecane composite was 1.25 W·m^−^^1^·K^−^^1^, which is more than eight times to that of pure hexadecane [21].

Based upon the predecessors’ studies, three aspects of building thermal storage material should be further researched: latent heat storage materials with suitable phase temperature and high latent heat, supporting material with excellent performance, and application of enhanced heat transfer technology. The eutectic mixtures of fatty acid-alcohol have suitable phase change temperature, high latent heat, lower price, and wide sources from which to be obtained, which can be potential thermal storage materials for building energy storage. However, there was little literature to study them as thermal energy storage materials in form-stable PCMs. The purpose of paper is to study thermal physical and chemical properties of a series of form-stable PCMs, which consist of latent heat storage materials [tetradecanol-decanoic acid (TD-CA) eutectic mixture, tetradecanol-dodecanic acid (TD-LA) eutectic mixture, tetradecanol-tetradecanoic acid (TD-MA) eutectic mixture], and HDPE-EVA alloy as supporting materials. These form-stable PCMs were produced by a fusion method. Following, thermal properties, morphology characterization, and chemical compatibility of form-stable PCMs were determined by DSC, scanning electron microscopy (SEM), and Fourier-transform infrared (FI-IR), respectively. Thermal stability was explored by the accelerated melting-solidification experiment. Furthermore, endothermic and exothermal experiment between these form-stable PCMs and corresponding composites with AP was developed to verify the improvement of heat transfer effect when introducing AP into form-stable PCMs.

## 2. Experimental

### 2.1. Materials

CA, LA, MA, and TD were supplied by Tianjin Guangfu Fine Chemical Research Institute and their purity was higher than 99.0%. Their thermal properties are listed in Table 1. Both supporting material (HDPE, EVA) and heat transfer enhancement material (AP) were obtained from Tianjin Le Tai Chemical Co., Ltd. (Tianjin, China).

**Table 1:** Properties of latent heat storage materials.

Material	Temperature (°C)		Latent heat (J/g)	
	Melting	Solidification	Melting	Solidification
CA	30.20	27.90	142.70	145.10
LA	43.20	40.30	177.70	180.40
MA	52.10	49.50	190.00	193.20
TD	37.00	36.80	207.30	222.00

### 2.2. Preparation of Form-Stable PCMs

The binary fatty acid/alcohol is considered as a balanced two-component solid-liquid material and the eutectic point is phase change point of binary mixture. Through the thermodynamic calculation of phase diagrams, the mass percentage, and phase change temperature of eutectic mixtures can be calculated by Equations (1) and (2) [26]:

(1) $$\ln x_{a}=\frac{\Delta_{S}^{l}H_{a}}{R}(\frac{1}{T_{a}}-\frac{1}{T_{f}})$$

(2) $$\ln(1-x_{a})=\frac{\Delta_{S}^{l}H_{b}}{R}(\frac{1}{T_{b}}-\frac{1}{T_{f}})$$

where $x_{a}$ and $T_{f}$ are the molar fraction of A in mixture and melting temperature of mixture respectively, $\Delta_{S}^{l}H$ and *T* are latent heat of fusion and melting temperature of pure material, which the subscript of a and b represent two materials, respectively. *R* is the gas constant which is equal to 8.315 J·k^−^^1^·mol^−^^1^. For certain types of material in binary mixed solution, $T_{a}$, $T_{b}$, $\Delta_{S}^{l}H_{a}$ and $\Delta_{S}^{l}H_{b}$ are constant, so $x_{a}$ and $T_{f}$ can be calculated. The mass percentage of four eutectic mixtures was shown in Table 2.

**Table 2:** The mass percentage of eutectic mixtures, which are calculated by the above equations.

Fatty alcohol	Fatty acids	Mass percentage (wt %)
TD	CA	38.00–62.00
TD	LA	53.60–46.40
TD	MA	71.84–28.16

In tradition, fatty acids are a kind of pungent odor organic materials, while fatty alcohols with aromatic odor are part of cosmetics, food, and industrial solvents. Mixed them together, the pungent odor can be diluted. Moreover formed eutectic mixture of acid and alcohol as form-stable PCM, the problem of pungent odor of fatty acids hampering its application can be solved. Moreover in order to improve flammability, flame retardants, comprised of ammonium polyphosate and pentaerythritol as flame retardant additives have been added to the form-stable PCMs as carbonization catalyst and metallic oxide were used in form-stable PCM. Cai [13,18,19] have proved that the form-stable PCM with flame retardant produces a larger amount of char residue at 800 °C, which improve the effect of flame retardant on form-stable PCM.

**Figure 1:**
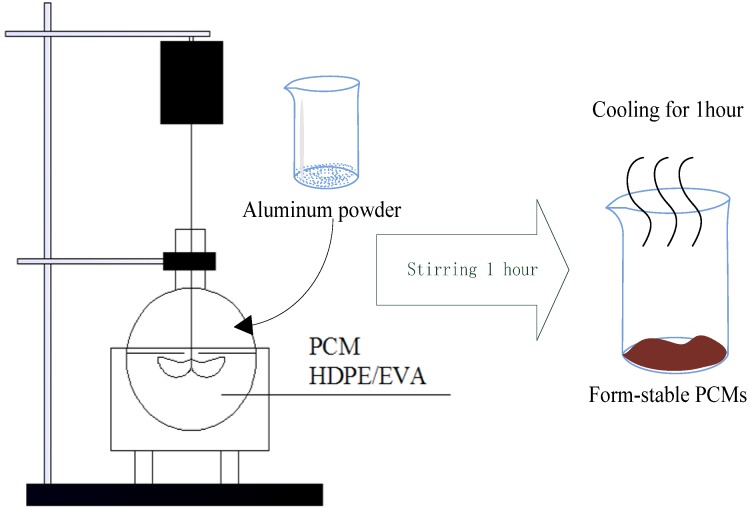
The preparation process of form-stable phase change materials (PCMs) by fusion method.

The melt-mixing method was adopted in this form-stable PCM preparation. The preparation process of form-stable PCMs was shown in Figure 1. The detail preparation process was listed as follow: at first, organic acids and TD were weighted in given proportion, mixed, then melted together in an oil-bath under 70 °C for 20 min. Then introduced weighted HDPE and EVA, these mixtures were melted and stirred in oil-bath with constant temperature of 150 °C about 40 min. The mass ratio of HDPE-EVA was fixed as 1:1. Ammonium polyphosate and pentaerythritol, as flame retardant additive, was added to the form-stable PCMs as Cai [13]. To confirm the maximum encapsulation capacity of HDPE-EVA alloy without any leakage when form-stable PCM undergoes melting process, five types of mass ratio were identified in Table 3.

**Table 3:** The mass ratio of form-stable phase change materials (PCMs).

PCMs	Supporting material	Mass percentage (wt %)
Eutectic mixtures of fatty acid(CA,LA, MA) and fatty alcohol (TD)	HDPE-EVA	60.00%–40.00%
		65.00%–35.00%
		70.00%–30.00%
		75.00%–25.00%
		80.00%–20.00%

**Figure 2:**
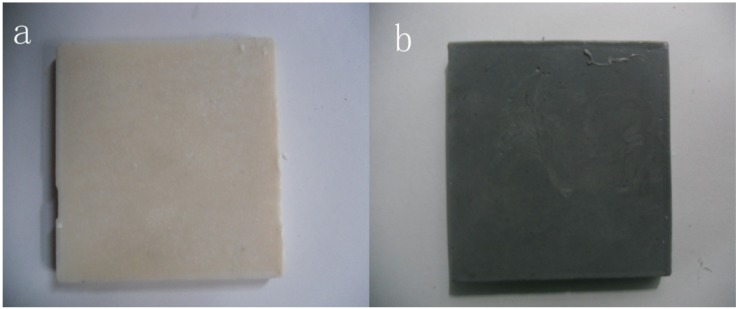
(**a**) Samples of form-stable PCM; (**b**) Samples of form-stable PCM with AP.

The weight of AP accounted for 10% of total mass of form-stable PCM. A corrosive test of aluminum powder has been made. The result indicated fatty acid did not corrode aluminum. The samples of form-stable PCM with/without AP were presented in Figure 2. The white one was form-stable PCM without AP and black one was form-stable PCM with AP.

### 2.3. Analysis Method

The surface morphology of form-stable PCMs with/without AP was determined by SEM. Two kinds of observation scales of 50 and 20 μm were detected. SEM images were obtained on a PHILIPS XL30ESEM microscope (PHILIPS, Amsterdam, The Netherlands).

FT-IR spectral analysis [Nicolet 6700 FT-IR Spectrometer (Thermo Scientific, Waltham, MA, USA)] was done to explore whether there was chemical reaction between different components of form-stable PCMs. Infrared spectrum of eutectic mixtures, HDPE-EVA polymer and their composites were taken on a KBr disc at a frequency of 400–4000 cm^−1^, respectively.

The thermal properties such as phase change temperature and latent heat of PCMs and their composites were analyzed by DSC [204 F1 Phoenix (NETZSCH, Lichfield, Germany)] at a heating rate of 1 °C/min between 5 and 50 °C in a purified nitrogen atmosphere.

The thermal cycling test was carried to analyzed thermal stability of form-stable PCMs after the accelerated endothermic-exothermal cycling. The thermal properties of these form-stable PCMs after accelerated thermal cycling were measured by DSC.

### 2.4. Thermal Cycling Test

**Figure 3:**
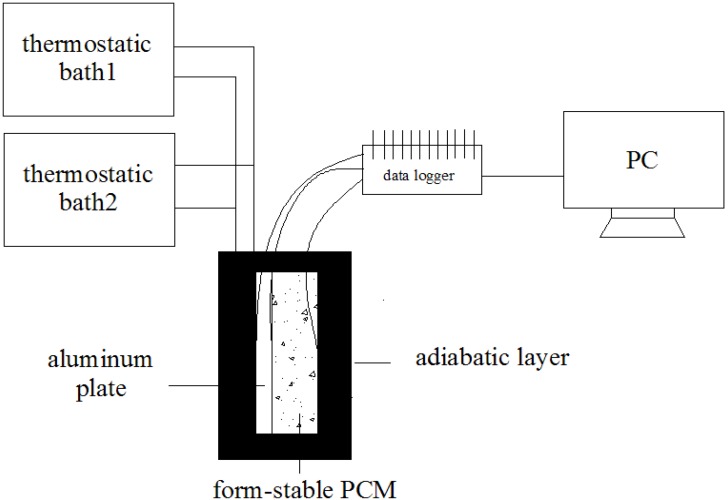
Thermal storage and release process experimental set-up.

The thermal transfer effect experiment between form-stable PCM with/without AP was tested by a constant temperature test bench. Figure 3 was an experimental set-up sketch. This experiment, which was composed of three parts: cold/heat source, the testing part and the data acquisition part. The cold/heat source was thermostatic baths where thermostatic bath1 controlled the temperature in 45 °C for endothermic process and another was set on 10 °C for exothermal process. Water was circulated between thermostatic baths and aluminum hollow core slab. The testing part was packaged by adiabatic material. The temperature and heat flux of form-stable PCM and aluminum plate were detected by thermocouples and heat flow meters. The final part was PC-based data acquisition unit MX100 (Yokogawa), which automatically recorded data at time intervals of five seconds.

## 3. Results and Discussion

### 3.1. Selection of Proper Mass Percentage of Different Form-Stable PCMs

As thermal storage material, latent heat of form-stable PCM is an important indicator to determine whether this composite can be used or not [15]. The larger mass percentage of PCM is contained in the form-stable PCM, the larger latent heat of this form-stable PCM can be obtained. However if the mass percentage of PCM exceeds the limit, which HDPE-EVA polymer can bear, PCM will leak in liquid state inevitably. To obtain the maximum encapsulation capacity of different kinds of form-stable PCMs, cyclic endothermic and exothermal experiment was carried out. Every form-stable PCM underwent 50 times thermal cycles of endothermic process at constant temperature of 45 °C for 1.5 h and exothermic process at constant temperature of 10 °C for 30 min. The mass leakage percent was defined as:

(3) $$\varphi_{leakage}(wt\%)=\frac{M_{1}-M_{2}}{M_{1}}\times 100\%$$

where $M_{1}$ and $M_{2}$ stand for form-stable PCM mass before thermal cycle process and at the end of every thermal cycle process respectively. As the above, analysis, total mass leakage percent determined the latent heat of form-stable PCM, which determined the thermal storage effect, so total mass leakage percent was considered as an important factor to choose suitable encapsulation proportion. In theory, when HDPE-EVA alloy increases by 5%, the theoretical latent heat reduces about 7%. In order to keep high latent heat, the mass leakage percent is controlled in less than 5%. Table 4 indicates total mass leakage percent of different kinds and different mass percentage of form-stable PCMs. The slight liquid leakage always occurs in the first few cycles. After that there was no more liquid leakage. The data in black frame are complied with the above requirement. Compared the mass leakage percent between different mass ratios of FS TD-CA PCM, it can be found total mass leakage percent decreased with the HDPE-EVA polymer mass increase. When the mass percentage of PCM accounted for 80% in form-stable PCM, the form-stable PCM broke after endothermic and exothermal cycle process. Based on optimization of latent heat and mass leakage percent, the composite with 70 wt % TD-CA and 30 wt % HDPE-EVA polymer, composite with 65 wt %TD-LA and 35 wt % HDPE-EVA polymer and composite with 60 wt %TD-MA and 40 wt % HDPE-EVA polymer were selected as potential building thermal storage material, which is studied in the following parts. Contrasting between those selected form-stable PCMs, it could be found that the maximum mass percents of different PCMs in these composites without leakage were different. That means the packaging effect of HDPE-EVA polymer for different PCMs varied greatly. In process of preparation of eutectic mixture and form-stable PCM under melt-mixing method, there was rarely chemical reaction among those materials [5,12,16]. CA, LA and MA were main different part of PCM. The functional groups and chemical structure of these three fatty acids are similar. The main different between them are molecular weight and molecular size. With the reduction of the molecular weight of PCM, the mass leakage of form-stable PCMs reduces.

**Table 4:** Mass leakage percent of different kinds of form-stable PCMs.

Form-stable PCMs and corresponding percent	60%–40%	65%–35%	70%–30%	75%–25%	80%–20%
FS TD-CA PCM	1.31%	2.60%	4.45%	7.10%	-
FS TD-LA PCM	2.38%	4.23%	7.57%	10.10%	-
FS TD-MA PCM	3.12%	5.61%	8.31%	11.28%	-

Notes: FS TD-CA PCM stands for form-stable TD-CA PCM; FS TD-LA PCM stands for form-stable TD-LA PCM; FS TD-MA PCM stands for form-stable TD-MA PCM.

### 3.2. Morphology Characterization of the Form-Stable PCMs

**Figure 4:**
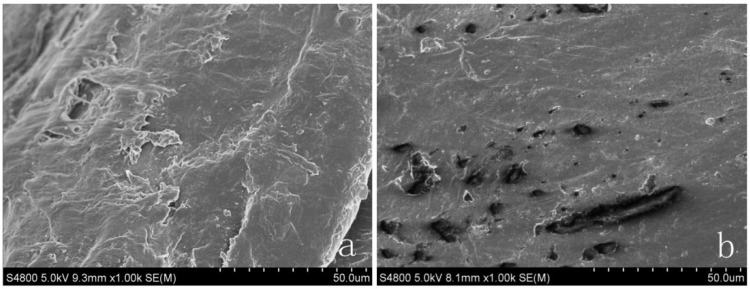
Morphological appearance of supporting materials (**a**) SEM image of HDPE (500×); (**b**) SEM image of EVA (500×).

**Figure 5:**
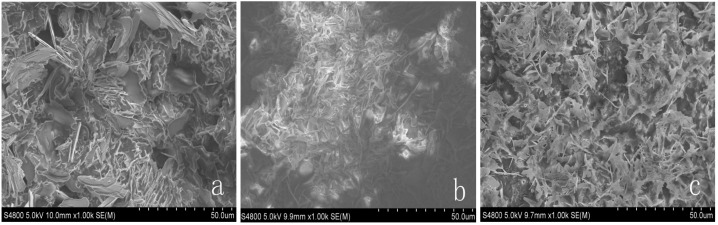
(**a**) SEM images of FS TD-CA PCM (500×); (**b**) SEM images of FS TD-LA (500×); (**c**) SEM images of FS TD-MA PCM (500×).

The surface morphology and microstructures of HDPE, EVA, HDPE-EVA polymer, and form-stable PCMs were surveyed by SEM. Figure 4 indicates surface morphology image of HDPE (a) and EVA (b). As seen from Figure 4, HDPE is layered structure while there are several holes in surface of EVA. Both of them are relatively compact structures. In the melting process, ductile EVA melt then filled in HDPE. In Figure 5, microstructures of three form-stable PCMs are listed. The features of Figure 5 are almost the same. The light and dark parts are HDPE-EVA polymer and PCM within the form-stable PCMs respectively. It was observed the HDPE-EVA polymer forms the cross structure, which replaces layer structure after high temperature melting and mixing process. This structure effectively maintained the form of form-stable PCMs and prevented leakage of PCM when undergoing the melting process.

### 3.3. FT-IR Spectroscopy Analysis

**Figure 6:**
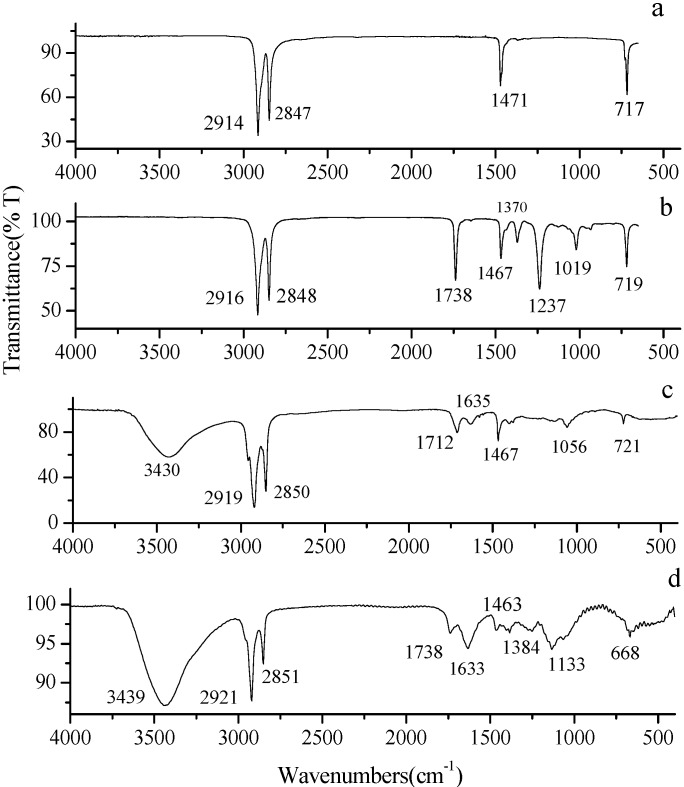
FT-IR spectra analysis. (**a**) FT-IR spectra of HDPE; (**b**) FT-IR spectra of EVA; (**c**) FT-IR spectra of eutectic mixture of TD-MA; (**d**) FT-IR spectra of FS TD-MA PCM.

The chemical compatibility between PCMs, HDPE, and EVA were tested by FT-IR spectroscopy. As basic chemical component of three composites were almost same, FS TD-MA PCM was selected as a sample. The FT-IR absorption spectrum of HDPE, EVA, TD-MA eutectic mixture, and FS TD-MA PCM are presented in Figure 6.

Figure 6c stands for FT-IR spectrum of TD-MA eutectic mixture, alcohols have characteristic absorption peaks associated with both the O–H and the C–O stretching vibrations. The O–H and C–O stretch appear in the region 3500–3200 cm^−1^ and 1260–1050 cm^−1^, respectively. Unlike O–H stretch band of alcohols observed, O–H stretch band of acids shows up in the region of 2500–3300 cm^−1^, which is the same region as C–H stretching bands of both alkyl groups. Analyzed as follows, the peak at 3430 cm^−1^ are O–H stretching vibrations of TD. The peak at 2919 cm^−1^ and 2850 cm^−1^ are determined as O–H stretching vibrations of MA. The absorption peak of C=O stretching band appears at 1712 and 1635 cm^−1^. The peak detected at 1467 cm^−1^ is the –CH2 bending peaks. The peaks at 1056 and at 720 cm^−1^ represent a C–O stretching band of TD and bending of aliphatic chain of MA, respectively. Figure 6a indicates the FT-IR spectrum of HDPE. The absorption peak of C–H stretching vibrations is indicated at 2914 and 2847 cm^−1^. The peaks at 1471 and 717 cm^−1^ signify bending of –CH2 peaks and =CH peaks, respectively. In Figure 6b, the peaks at 1738 and 1019 cm^−1^ imply stretching vibration of C=O group and peak at 1237 cm^−1^ is C–O group. Compared to the FT-IR spectra of Figure 6, it can be found that there is a peak at 1133 cm^−^^1^ in FS TD-MA PCM, which does not appear in other three materials. However, the peaks at 1019 and 1237 cm^−^^1^ of HDPE and the peak at 1056 cm^−^^1^ of TD-MA eutectic mixture disappears in the FT-IR spectrum of FSTD-MA PCM. It is a slight peak shift phenomenon since interaction force of chemical bond [27]. Figure 6 indicates that the FT-IR spectrum of FS TD-MA PCM contained almost peaks of TD-MA eutectic mixture, HDPE and EVA, which means there was no chemical interaction between PCM and HDPE-EVA polymer. The conclusion is also applicable to FS TD-CA PCM and FS TD-LA PCM.

### 3.4. Thermal Properties of PCMs and Their Composites

**Figure 7:**
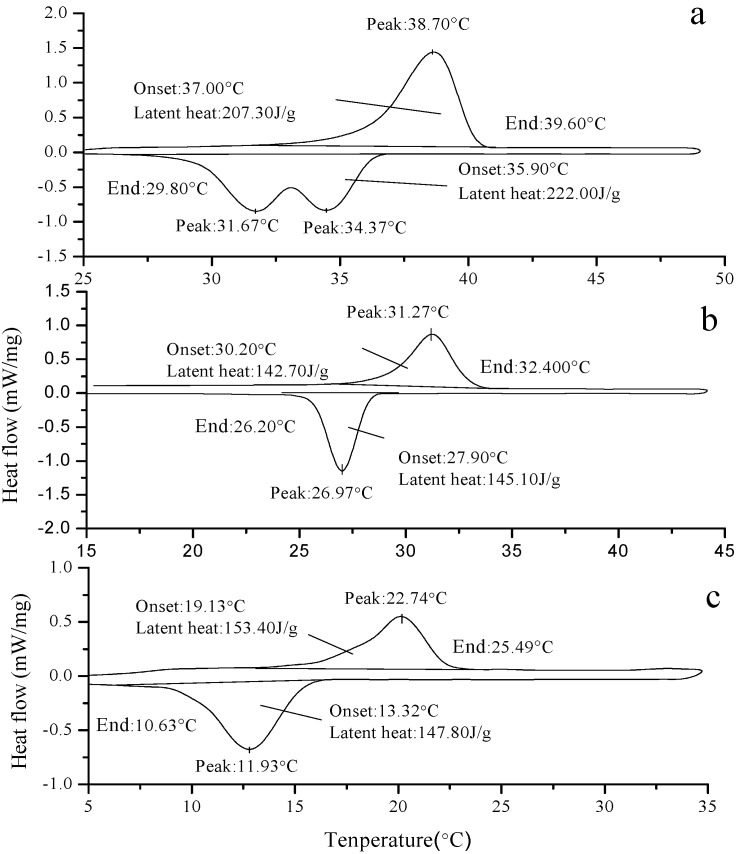
The result of onset temperatures, peak temperatures and latent heat. (**a**) DSC curve of TD; (**b**) DSC curve of CA; (**c**) DSC curve of TD-CA eutectic mixture.

Thermal properties of PCM and their composites were analyzed by DSC. Figure 7 describes melting and solidification curves of TD (Figure 7a), CA (Figure 7b), and TD-CA eutectic mixture (Figure 7c).The onset temperature, peak temperature, end temperature, and heat latent of them are listed in Table 1 and Table 5 respectively. The DSC curves show only one endothermic peak in all the melting curves of these three PCMs, while two continuous exothermic peaks in TD solidification curves are clearly observed in Figure 7a. The higher temperature exothermic peak is solid-liquid phase change exothermic peak. Another exothermic peak is solid-solid phase change as rotator phase α→ ordered phase γ [9]. As seen from Figure 7, eutectic mixture eliminate demixing and segregation of TD, which improved the chemical stability and latent heat utilization than muilt-peak phase change process. Gandolfo [9] studied mixtures of stearic acid and steary alcohol and got the same result. It also can be found that the smaller phase change radius of TD-CA than the phase change radius of TD made it better for building thermal storage. In other hand, TD can effectively eliminate the pungent odor of CA.

**Table 5:** Thermal properties of PCMs and their composites.

Materials	Melting				Solidification			
	Onset temperature (°C)	Peak temperature (°C)	End temperature (°C)	Enthalpy (J/g)	Onset temperature (°C)	Peak temperature (°C)	End temperature (°C)	Enthalpy (J/g)
TD-CA	19.13	22.74	25.49	153.40	13.32	11.93	10.63	147.80
FS TD-CA PCM	18.90	21.68	24.24	100.50	14.16	12.20	9.18	99.70
TD-LA	24.40	26.59	27.91	162.70	24.43	21.94	18.65	146.80
FS TD-LA PCM	24.53	27.64	30.18	90.20	24.92	22.85	19.88	88.70
TD-MA	34.45	36.46	38.05	208.00	31.98	30.51	29.43	206.20
FS TD-MA PCM	33.15	35.08	37.75	128.60	30.72	29.09	27.63	125.70

In summary, eutectic mixture of fatty acid and alcohol not only obtains a lower phase change temperature to achieve the goal of building thermal storage, but also makes up fatty acid and alcohol’s deficiencies, such as a larger phase change radius and pungent odor.

**Figure 8:**
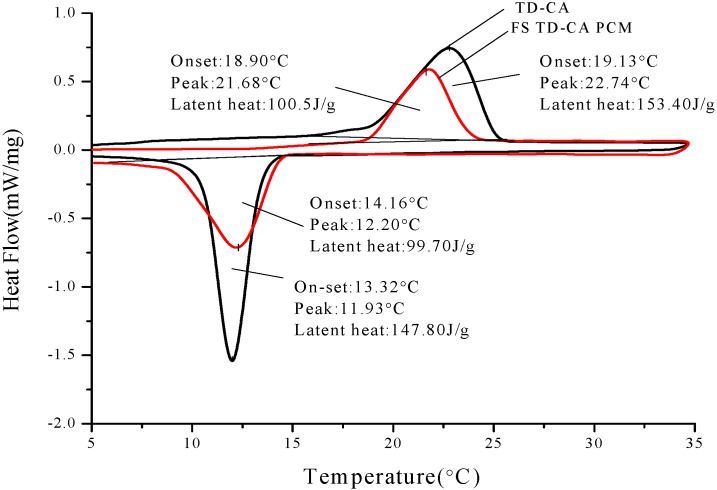
DSC curves of TD-CA eutectic mixture and FS TD-CA PCM. Their onset temperatures, peak temperatures and latent heat are present.

**Figure 9:**
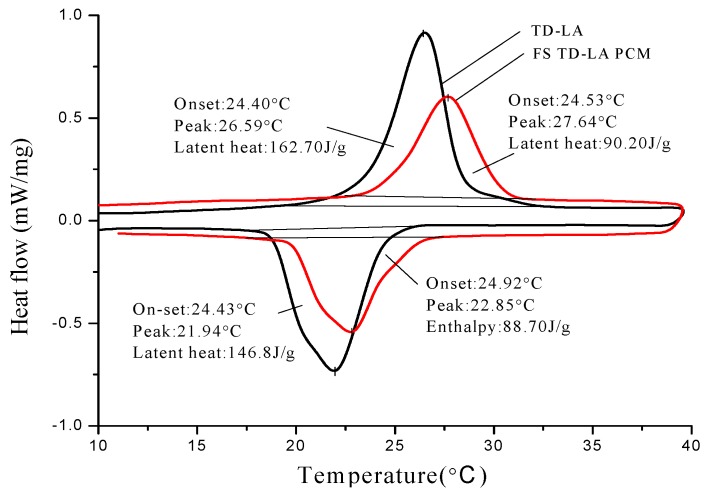
DSC curves of TD-LA eutectic mixture and FS TD-LA PCM. Their onset temperatures, peak temperatures, and latent heat are present.

**Figure 10:**
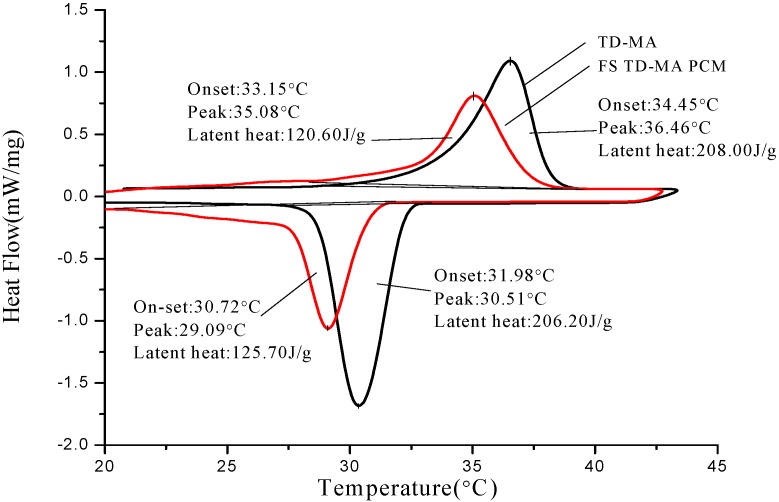
DSC curves of TD-MA eutectic mixture and FS TD-MA PCM. Their onset temperatures, peak temperatures and latent heat are present.

The thermal properties analysis of PCMs and corresponding form-stable PCMs are shown in Figure 8, Figure 9 and Figure 10. The onset phase change temperature is intersection temperature of the tangent of initial phase change point and the tangent of peak point. The end phase change temperature is intersection temperature of tangent of termination phase change point and tangent of peak point. The onset temperature, peak temperature, end temperature, and latent heat of melting and solidification are also listed in Table 5. As seen from Figure 8, onset melting temperature and peak melting temperature of TD-CA eutectic mixture are 19.13 and 22.74 °C, respectively. In addition, onset solidification temperature and peak solidification temperature of TD-CA eutectic mixture are 13.32 and 11.93 °C, respectively. For FS TD-CA PCM, both onset temperature of melting and solidification varies as −0.23 and 0.84 °C. There is a slight temperature deviation between pure PCM and form-stable PCM. This is because the three-dimensional structure of HDPE-EVA polymer confines the movement of PCM in microcosmic angle, which was verified by Sari [28]. Figure 9 and Figure 10 signify similar results as Figure 8. The melting/solidification onset temperature of FS TD-LA PCM is 24.53 °C/24.92 °C. The melting/solidification onset temperature of FS TD-MA PCM is 33.15 °C/30.72 °C. The onset temperature variations between different PCMs and corresponding form-stable PCMs are not equal because of the interaction force of different groups in different PCMs.

Figure 8, Figure 9 and Figure 10 also shows latent heat of different PCMs and form-stable PCMs. The melting/solidification heat of TD-CA eutectic mixture and FS TD-CA PCM are 153.4 J/g/147.80 J/g and 100.50 J/g/99.70 J/g, which decreased by 34.49% and 32.54%, respectively. The theoretical latent heat of form-stable PCM is calculated as the produce of the enthalpy and mass percentage of PCM. With this principle, theoretical latent heat of FS TD-CA PCM was 107.38 J/g in melting and 103.46 J/g in solidification, which was higher than measure value. The temperature of oil bath was set to 150 °C in the form-stable PCM preparation process. Under the combined action of high temperature and moderate agitation, a small amount of PCMs volatilized, which caused the actual latent heat, lower than the calculation value. The melting/solidification latent heat of FS TD-LA PCM and FS TD-MA PCM were 90.20 J/g/88.70 J/g and 128.60 J/g/125.70 J/g, which conforms to the law of latent heat loss.

Three form-stable PCMs are potential building thermal storage materials on account of lager latent heat and appropriate phase change temperature within building environment temperature (10–40 °C). The phase change temperature of FS TD-LA PCM is close to indoor comfortable temperature, so it can be applied in the inner side of building envelop. The FS TD-MA PCM can be used in the outer side of the building envelope. The phase change temperature of FS TD-CA PCM is relatively low, so it should be used in combination with heat/cold source.

### 3.5. Thermal Stability of Form-Stable PCMs

**Figure 11:**
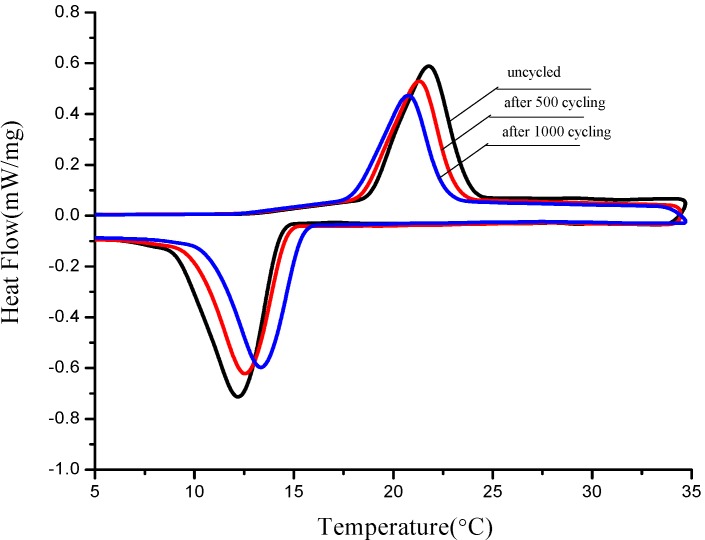
DSC curves of FS TD-CA PCM before cycling, after 500 and 1000 cycling.

**Figure 12:**
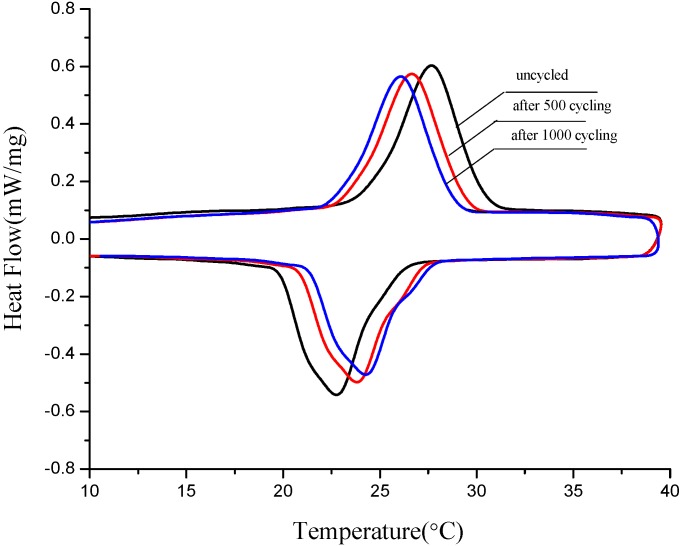
DSC curves of FS TD-LA PCM before cycling, after 500 and 1000 cycling.

**Figure 13:**
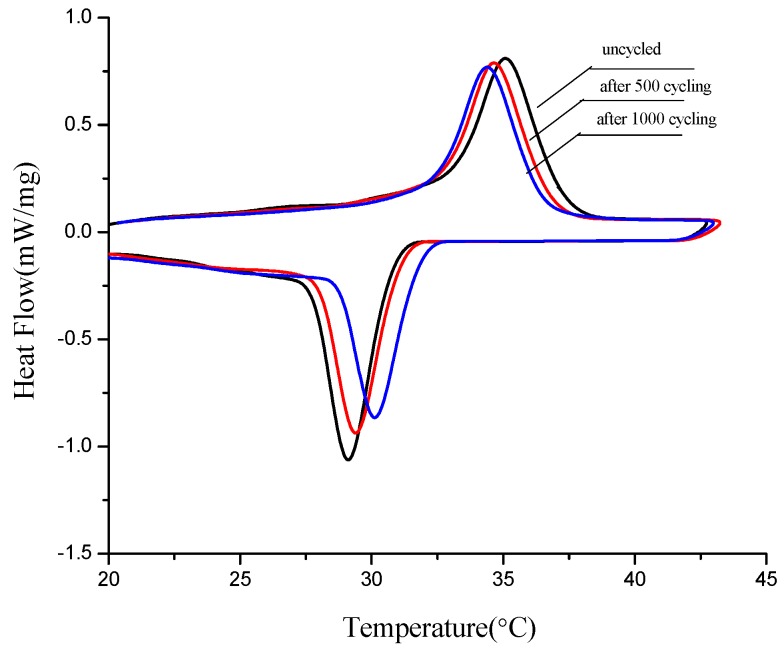
DSC curves of FS TD-MA PCM before cycling, after 500 and 1000 cycling.

Thermal stability is one of factors to determine whether the materials can be used or not in the long term. Therefore thermal cycling tests of three form-stable PCMs were developed after 500 and 1000 melting/solidification cycles, then those samples were detected by DSC. Figure 11, Figure 12 and Figure 13 are thermal curves of three form-stable PCMs before and after thermal cycling and their thermal properties are given in Table 6. After thermal cycling, the melting/solidification temperature of FS TD-CA PCM changed as −0.47 °C/0.5 °C and −0.78 °C/1.24 °C, after 500 and 1000 cycles, respectively. The melting/solidification latent heat of FS TD-CA PCM decreased 1.79%/3.31% and 4.17%/5.22%, after 500 and 1000 cycles, respectively. This slight change of phase temperature and latent heat don’t affect the availability for building thermal storage. As seen from Figure 12 and Figure 13, and Table 6, this slight change can be also found in FS TD--LA PCM and FS TD-MA PCM. It can be concluded that these three form-stable PCM shows good thermal stability after long-term cycles, which ensure these materials can be use for a long time as building thermal materials.

**Table 6:** Thermal properties of form-stable phase change materials (FSPCMs) before and after cycling.

Materials		Melting			Solidification		
		Onset temperature (°C)	End temperature (°C)	Enthalpy (J/g)	Onset temperature (°C)	End temperature (°C)	Enthalpy (J/g)
FS TD-CA PCM	0 cycling	18.90	24.24	100.50	14.16	9.18	99.70
	500 cycling	18.47	23.34	98.70	14.66	9.53	96.40
	1000 cycling	18.12	22.90	96.30	15.40	10.18	94.50
FS TD-LA PCM	0 cycling	24.53	30.18	90.20	24.92	19.88	88.70
	500 cycling	23.62	29.69	87.60.	25.93	20.67	86.40
	1000 cycling	23.22	29.28	85.70	26.23	21.12	85.10
FS TD-MA PCM	0 cycling	33.15	37.75	128.60	30.72	27.63	125.70
	500 cycling	32.81	37.28	125.30	31.11	28.00	123.40
	1000 cycling	32.65	37.04	123.50	31.72	28.55	121.70

### 3.6. Thermal Storage and Release Performance of the Form-Stable PCMs

**Figure 14:**
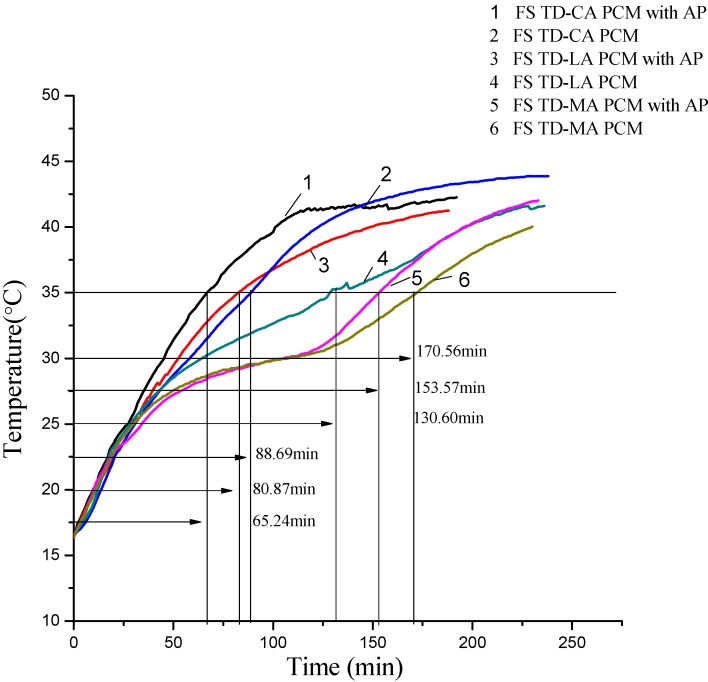
Comparison diagram of melting temperature curves between FS TD-CA PCM, FS TD-LA PCM, and FS TD-MA PCM with/without AP.

**Figure 15:**
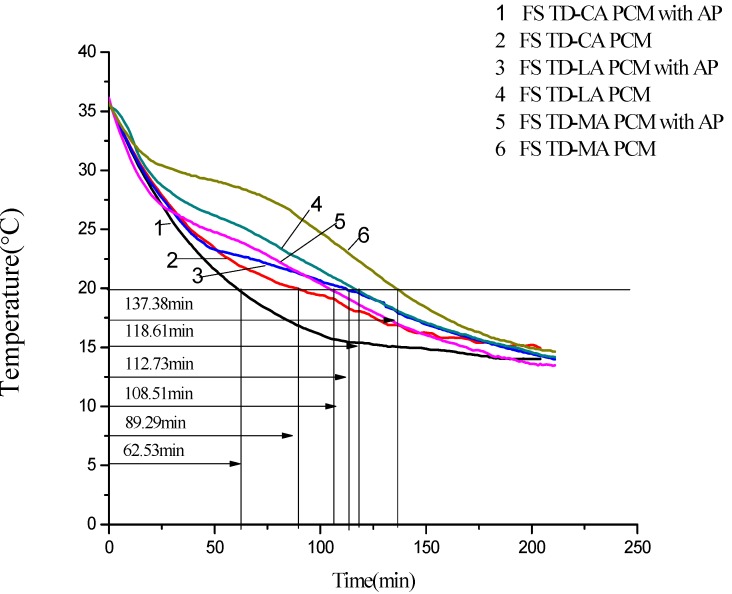
Comparison diagram of solidification temperature curves between FS TD-CA PCM, FS TD-LA PCM, and FS TD-MA PCM with/without AP.

Thermal storage and release rates are important indexes to reflect heat transfer efficiency, which was indicated by melting and solidification temperature curses of form-stable PCM. The melting and solidification processes of FS TD-CA PCM with/without AP, FS TD-LA PCM with/without AP, and FS TD-MA PCM with/without AP were displayed in Figure 14 and Figure 15. As seen from Figure 14, the melting time of FS TD-CA PCM, FS TD-LA PCM, and FS TD-MA PCM with/without AP are 65.24 min/80.87 min, 88.69 min/130.60 min and 153.57 min/170.56 min, respectively, heated from 16.00 to 35.00 °C. The melting time decreased about 19.33%, 32.09% and 9.96%. The solidification time were shortened from 89.29 to 62.53 min after embedding AP into FS TD-CA PCM at the temperature from 35.80 to 20.00 °C. Similarly, as seen from Figure 15, the solidification time of other two form-stable PCMs and form-stable PCMs with AP (FSPCM/AP) are 108.51 min/112.73 min, 118.61 min/137.38 min, respectively. In the two figures above, it can be found that there is an apparent heat transfer enhancement effect by introducing AP into these three form-stable PCMs, which means that adding AP into form-stable PCMs is a useful method to improve the whole thermal conductivity of form-stable PCMs.

## 4. Conclusions

In this paper, three novel form-stable PCMs based on eutectic mixtures of fatty acid-TD and HDPE-EVA polymer were prepared for thermal storage in building. The thermal properties, surface morphology, thermal reliability, chemical stability, and thermal storage/release performance were determined by DSC, SEM, FT-IR, and melting/solidification cycling test. The maximum mass percentages of PCMs in three form-stable PCMs without leakage were 70 wt % (TD-CA), 65 wt % (TD-LA) and 60 wt % (TD-MA). Compared with maximum mass percentages of three form-stable PCMs, it can be found mass leakage decreased with molecular weight of PCM decreasing. The onset melting/solidification temperature and latent heat of form-stable PCMs were measured as 19.13 °C/13.32 °C/100.50 J/g/99.70 J/g (TD-CA), 24.53 °C/24.92 °C/90.20 J/g/88.70 J/g (TD-LA) and 33.15 °C/30.72 °C/128.60 J/g/125.70 J/g (TD-MA). The results of SEM, FT-IR, and 1000 times thermal cycling tests are showed that three form-stable PCMs had good thermal reliability, chemical stability and thermal stability. AP additive improve the thermal storage/release performance of three form-stable PCMs and the maximum thermal storage/release rates were increased almost 32.09% and 29.96%. Based on the aforementioned results, these three form-stable PCMs can be considered as potential building thermal energy storage material to keep comfort indoor environment and save energy.

## References

[1] Farid M.M., Khudhair A.M., Razack S.A.K., Al-Hallaj S. (2004). A review on phase change energy storage: Materials and applications. Energy Conv. Manag..

[2] Tyagi V.V., Buddhi D. (2007). PCM thermal storage in buildings: A state of art. Renew. Sust. Energ. Rev..

[3] Kuznik F., David D., Johannes K., Roux J.J. (2011). A review on phase change materials integrated in building walls. Renew. Sust. Energ. Rev..

[4] Sharma A., Tyagi V.V., Chen C.R., Buddhi D. (2009). Review on thermal energy storage with phase change materials and applications. Renew. Sust. Energ. Rev..

[5] Sari A., Kaygusuz K. (2002). Thermal performance of a eutectic mixture of lauric and stearic acids as PCM encapsulated in the annulus of two concentric pipes. Sol. Energy.

[6] Sari A., Karaipekli A. (2009). Preparation, thermal properties and thermal reliability of palmitic acid/expanded graphite composite as form-stable PCM for thermal energy storage. Sol. Energy Mater. Sol. Cells.

[7] Shilei L., Neng Z., Feng G.H. (2006). Eutectic mixtures of capric acid and lauric acid applied in building wallboards for heat energy storage. Energy Build..

[8] Wang Y., Xia T.D., Feng H.X., Zhang H. (2011). Stearic acid/polymethylmethacrylate composite as form-stable phase change materials for latent heat thermal energy storage. Renew. Energy.

[9] Gandolfo F.G., Bot A., Floter E. (2003). Phase diagram of mixtures of stearic acid and stearyl alcohol. Thermochim. Acta.

[10] Zuo J.G., Li W.Z., Weng L.D. (2011). Thermal performance of caprylic acid/1-dodecanol eutectic mixture as phase change material (PCM). Energy Build..

[11] Zeng J.L., Cao Z., Yang D.W., Xu F., Sun L.X., Zhang L., Zhang X.F. (2009). Phase diagram of palmitic acid-tetradecanol mixtures obtained by DSC experiments. J. Therm. Anal. Calorim..

[12] Zuo J.G., Li W.Z., Weng L.D. (2011). Thermal properties of lauric acid/1-tetradecanol binary system for energy storage. Appl. Therm. Eng..

[13] Cai Y.B., Hu Y., Song L., Lu H.D., Chen Z.Y., Fan W.C. (2006). Preparation and characterizations of HDPE-EVA alloy/OMT nanocomposites/paraffin compounds as a shape stabilized phase change thermal energy storage material. Thermochim. Acta.

[14] Kenisarin M.M., Kenisarina K.M. (2012). Form-stable phase change materials for thermal energy storage. Renew. Sust. Energ. Rev..

[15] Mei D.D., Zhang B., Liu R.C., Zhang Y.T., Liu J.D. (2011). Preparation of capric acid/halloysite nanotube composite as form-stable phase change material for thermal energy storage. Sol. Energy Mater. Sol. Cells.

[16] Sari A. (2004). Form-stable paraffin/high density polyethylene composites as solid-liquid phase change material for thermal energy storage: Preparation and thermal properties. Energy Conv. Manag..

[17] Inaba H., Tu P. (1997). Evaluation of thermophysical characteristics on shape-stabilized paraffin as a solid–liquid phase-change material. Heat Mass Transf..

[18] Cai Y.B., Hu Y., Song L., Kong Q.H., Yang R., Zhang Y.P., Chen Z.Y., Fan W.C. (2007). Preparation and flammability of high density polyethylene/paraffin/organophilic montmorillonite hybrids as a form stable phase change material. Energy Conv. Manag..

[19] Cai Y.B., Wei Q.F., Huang F.L., Gao W.D. (2008). Preparation and properties studies of halogen-free flame retardant form-stable phase change materials based on paraffin/high density polyethylene composites. Appl. Energy.

[20] Liu M., Saman W., Bruno F. (2012). Review on storage materials and thermal performance enhancement techniques for high temperature phase change thermal storage systems. Renew. Sustain. Energy Rev..

[21] Darkwa J., Zhou T. (2011). Enhanced laminated composite phase change material for energy storage. Energy Conv. Manag..

[22] Ho C.J., Gao J.Y. (2009). Preparation and thermophysical properties of nanoparticle-in-paraffin emulsion as phase change material. Int. Commun. Heat Mass Transf..

[23] Zeng J.L., Sun L.X., Xu F., Tan Z.C., Zhang Z.H., Zhang J., Zhang T. (2007). Study of a PCM based energy storage system containing Ag nanoparticles. J. Therm. Anal. Calorim..

[24] Sari A., Karaipekli A. (2007). Thermal conductivity and latent heat thermal energy storage characteristics of paraffin/expanded graphite composite as phase change material. Appl. Therm. Eng..

[25] Mettawee E.B.S., Assassa G.M.R. (2007). Thermal conductivity enhancement in a latent heat storage system. Sol. Energy.

[26] Li M., Wu Z.S., Kao H.T. (2011). Study on preparation, structure and thermal energy storage property of capric-palmitic acid/attapulgite composite phase change materials. Appl. Energy.

[27] Sari A., Bicer A. (2012). Preparation and thermal energy storage properties of building material-based composites as novel form-stable PCMs. Energy Build..

[28] Kaygusuz K., Sari A. (2007). High density polyethylene/paraffin composites as form-stable phase change material for thermal energy storage. Energy Sources Part A Recovery Utili. Environ. Eff..

